# Functional Brain Activity Associated With Intermittent Rhythmic Delta/Theta Activity: A Transdiagnostic Electroencephalography–Functional Magnetic Resonance Imaging Resting-State Study

**DOI:** 10.1016/j.bpsgos.2025.100661

**Published:** 2025-11-29

**Authors:** Bernd Feige, Katharina von Zedtwitz, Isabelle Matteit, Andrea Schlump, Volker A. Coenen, Kathrin Nickel, Kimon Runge, Harald Prüss, Alexander Rau, Marco Reisert, Swantje Matthies, Katharina Domschke, Simon J. Maier, Ludger Tebartz van Elst, Dominique Endres

**Affiliations:** a Department of Psychiatry and Psychotherapy, Medical Center - University of Freiburg, Faculty of Medicine, University of Freiburg, Freiburg, Germany; b Department of Stereotactic and Functional Neurosurgery, Medical Center - University of Freiburg, Faculty of Medicine, University of Freiburg, Freiburg, Germany; c Department of Neurology and Experimental Neurology, Charité Universitätsmedizin Berlin, Berlin, Germany; d German Center for Neurodegenerative Diseases Berlin, Berlin, Germany; e Department of Neuroradiology, Medical Center - University of Freiburg, Faculty of Medicine, University of Freiburg, Freiburg, Germany; f Medical Physics, Department of Diagnostic and Interventional Radiology, Medical Center - University of Freiburg, Faculty of Medicine, University of Freiburg, Freiburg, Germany; g German Center for Mental Health, Partner Site Berlin/Potsdam, Berlin, Germany

**Keywords:** Autoimmune psychosis, Borderline personality disorder, EEG slowing, Epilepsy, Excitation, MREG

## Abstract

**Background:**

Intermittent rhythmic delta/theta activity (IRDA/IRTA) detected via electroencephalography (EEG) has been implicated in the pathophysiology of neuropsychiatric illnesses. Therefore, a combined EEG and functional magnetic resonance imaging (fMRI) approach was applied in a transdiagnostic group of patients with different causalities, i.e., autoimmune-mediated (in suspected autoimmune psychiatric syndromes [APS]) and primary psychiatric (borderline personality disorder [BPD]) causalities, as well as in healthy control (HC) participants, to characterize the brain regions functionally correlated with IRDA/IRTA.

**Methods:**

Overall, 135 EEG-fMRI datasets met the quality criteria, including 33 patients with suspected APS, 59 cases with BPD, and 43 HC participants. fMRI data were obtained using ultrafast MR encephalography and analyzed using AFNI. IRDA/IRTA events were separated from artifacts using independent component analysis and detected algorithmically. Brain regions (clusters) significantly correlated with IRDA/IRTA were first determined in all participants. Clusters occurring across all groups were classified as consensus areas. The groups were also analyzed individually, adding disease- or disorder-specific clusters not overlapping with the consensus areas.

**Results:**

Eleven consensus areas were identified across the 3 groups: 5 of them showed increased activity (Brodmann area [BA] 43-right [r], BA 2-left [l], BA 4-r, BA 18-r, BA 26/29/30-r), and 6 had reduced activity (BA 39-l, BA 10-l, BA 23-l, BA 19-l, BA 10-r, BA 18-l). The APS group showed 5 additional clusters, all with reduced activity (BAs 39-r, 1/3-r, 8-r, 4-l, 21-r). The BPD group showed one further cluster with increased activity (BA 17-l).

**Conclusions:**

In this study, IRDA/IRTA-related brain activity changes across the groups were identified, with excitatory brain activity especially in fronto-centro-temporal brain areas with similarities to the salience network. Additional disease- or disorder-specific changes were discovered in APS and BPD.

## Plain Language Summary

Intermittent rhythmic delta/theta activity (IRDA/IRTA) detected via electroencephalography (EEG) has been implicated in the pathophysiology of neuropsychiatric illnesses for decades but has been scarcely studied systematically. Therefore, a combined EEG and functional magnetic resonance imaging (fMRI) approach was used to analyze the resting state in a transdiagnostic group of patients with suspected autoimmune psychiatric syndromes (APS) (*n* = 33) and borderline personality disorder (BPD) (*n* = 59), as well as in healthy control (HC) participants (*n* = 43). Brain regions functionally correlated with IRDA/IRTA as well as group differences between patients with different causalities (i.e., autoimmune mediated vs. primary psychiatric) and HC participants were analyzed. A large fraction of transdiagnostic IRDA/IRTA-related brain activity changes was identified, i.e., 11 consensus areas across the 3 groups; 5 of them showed increased activity, and 6 areas had reduced activity. The activated consensus areas have similarities with the salience network as an integrating area. Excitatory EEG activity was also identified in the HC group; however, additional disease- or disorder-specific changes were only discovered in patients with APS and BPD.

The electroencephalography (EEG) phenomenon of intermittent rhythmic delta/theta activity (IRDA/IRTA) has been known for decades and repeatedly linked to the pathogenesis of neuropsychiatric disorders (1, 2, 3, 4, 5, 6, 7, 8, 9, 10). IRDA/IRTA is a transdiagnostically detectable finding identified in several neuropsychiatric disorders and, more rarely, in healthy control (HC) groups (6, 7, 8,11). IRDA and IRTA are both relatively easily recognizable phenomena in the clinical EEG. They represent rhythmically occurring intermittent, generalized slow EEG activity (delta or theta waves). IRDA/IRTA have repeatedly been interpreted as excitatory signs of neuronal network dysfunction. However, these EEG phenomena are understudied, and their functional significance remains largely unclear (6,7).

Similar EEG slowing has frequently been observed in autoimmune encephalitis (12,13). In the most frequent subtype, NMDA receptor encephalitis, EEG abnormalities were observed in 84% to 90% of all patients (14,15). Therefore, IRDA/IRTA events were included in the international consensus criteria for the psychiatric autoimmune encephalitis subtype of autoimmune psychosis (16), requiring clinical EEG as part of the diagnostic workup of patients with suspected autoimmune psychiatric syndromes (APS) (17,18). In our previous studies on various classical primary mental disorders, clinically visible IRDA/IRTA was most frequently detected in patients with borderline personality disorder (BPD) (6,11). A high prevalence of EEG changes in BPD has also been reported in other studies (19, 20, 21, 22, 23, 24). IRDA/IRTA can also be observed in epilepsies; for example, temporal IRDAs can be observed co-occurring with epileptiform discharges and interictally in temporal lobe epilepsy (25,26).

Automated EEG analysis techniques can be utilized to detect and quantify IRDA/IRTA, which is often difficult to identify visually or to discriminate from artifacts (7,8). Case reports documenting successful anticonvulsant treatment of patients with BPD or psychosis led to the development of the local area network inhibition (LANI) hypothesis (27,28). The LANI hypothesis proposes that local homeostatic inhibition as a consequence of hyperexcitability could lead to neuronal network dysfunction (27,28). Depending on the neuronal network involved, this could result in different clinical symptoms, e.g., dissociations in BPD through the involvement of networks including the anterior cingulum, or conversely, language- or memory-related symptoms with the involvement of temporal lobe networks in psychosis (28).

Simultaneous resting-state EEG–functional magnetic resonance imaging (EEG-fMRI) allows whole-brain mapping of functional correlates of IRDA/IRTA, as has been demonstrated for epilepsy-related events and spontaneous oscillatory modulations (29, 30, 31, 32, 33, 34, 35, 36, 37). A resting-state EEG/fMRI study with HC participants suggested a temporal association between IRDA and fMRI blood oxygen level–dependent (BOLD) signal alterations occurring during hyperventilation (38). Ultrafast MR encephalography (MREG) is particularly suitable for the localization of interictal events in epilepsies and possibly for detection of the functional correlates of IRDA/IRTA, in part because fast blood pulsation and respiratory as well as movement artifacts are adequately sampled and therefore can be individually controlled (39). Transdiagnostic investigations may have the potential to differentiate between varying pathophysiological causes (e.g., autoimmune-mediated vs. primary psychiatric etiology) of EEG phenomena.

The rationale of this project with a transdiagnostic approach was to investigate IRDA/IRTA-related brain activity changes using resting-state EEG and MREG fMRI. More specifically, the associations between IRDA and IRTA rates measured within the MRI scanner by EEG and the correlating functional clusters with reduced or increased brain activity—identified by EEG-fMRI—were analyzed. For this purpose, 2 patient groups with different causalities and a previous association with IRDA/IRTA were analyzed. More specifically, patients with APS with suspected secondary autoimmune-inflammatory etiology (18,40, 41, 42), patients with BPD with an assumed primary psychiatric etiology (43), and HC participants were compared. It was hypothesized that common brain areas would show IRDA/IRTA-related BOLD modulation across groups. Additionally, it was assumed that similar to the spike-related fMRI activations found for interictal spike-wave activity in epilepsies (30), brain activity changes can also be observed for IRDA/IRTA. Using an exploratory approach, it was tested whether there were additional disease- or disorder-specific, potentially pathological changes between patients with different etiologies.

## Methods and Materials

### Participants

This work was part of a multimodal imaging project with a focus on IRDA/IRTA funded by the German Research Foundation (Project No. 419859038). Ethical approval was received from the Ethics Committee of the Freiburg University Medical Center (EK-Freiburg: 209/18) in accordance to the principles of the Declaration of Helsinki. Before inclusion, all participants gave their written informed consent. The patient subgroups of this study cohort have been described in earlier publications (44, 45, 46).

### Patient and Control Group Assessment

**Figure 1:**
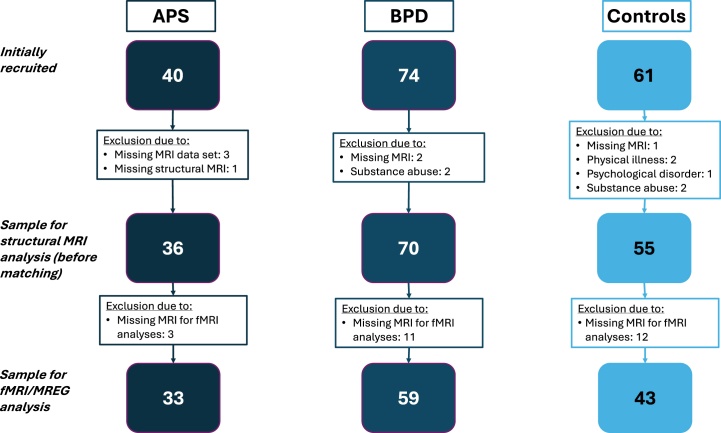
Recruitment flowchart. APS, autoimmune psychiatric syndromes; BPD, borderline personality disorder; EEG, electroencephalography; fMRI, functional magnetic resonance imaging; MREG, magnetic resonance encephalography; MRI, magnetic resonance imaging.

The entire recruitment flowchart is summarized in Figure 1. The process was described in detail in earlier publications (44, 45, 46).

The APS patient group consisted of adult patients (≥18 years) with suspected autoimmune psychosis spectrum syndromes, i.e., conditions with common autoantibody-associated autoimmune etiology. All patients tested positive for well-characterized neuronal/glial antibodies in cell-based assays or immunoassays (42), novel central nervous system (CNS) antibodies in a tissue-based assay on unfixed mouse brain slices (47,48), or well-characterized systemic antibodies (i.e., antinuclear antibodies measured on human embryonic kidney cells or thyroid antibodies and clear evidence of autoimmune brain involvement, with neuropsychiatric lupus or Hashimoto’s encephalopathy) (12,49,50). Clinically, the patients were selected following the APS concept (18,51,52), i.e., patients with different clinical manifestations (especially paranoid-hallucinatory schizophreniform psychosis, but also predominant affective, neurocognitive, or obsessive-compulsive syndromes) and stages of the disease were examined. Subgroups with predominantly schizophreniform psychoses and a subgroup with predominantly affective spectrum were defined. During the clinical workup, patients with APS underwent a broad diagnostic assessment, including routine MRI of the brain, EEG, cerebrospinal fluid (CSF) analysis, testing for CNS antibodies in serum and CSF, and, in several cases, [^18^F] fluorodeoxyglucose positron emission tomography of the brain (12,16, 17, 18). This corresponds to the recommended approach for this patient group (12,16, 17, 18). Psychopharmacological medication, anticonvulsants, or immunotherapies did not lead to exclusion. The detailed selection process is described elsewhere (44,46).

The BPD patient group comprised adult women (≥18 years) who were mainly recruited from our special dialectical behavior therapy ward for BPD (43) and were diagnosed by experienced senior psychiatrists. In addition, all patients met the diagnostic criteria for BPD in the Structured Clinical Interview for DSM-IV Axis II disorders. Patients with a comorbid diagnosis of schizophrenia, acute psychotic symptoms, bipolar disorder, or relevant neurological disorders, such as epilepsy, were excluded. Immunotherapies or anticonvulsants for treating epilepsies also led to exclusion. The details are described elsewhere (45).

The HC participants were >18 years old and had no current or lifetime mental illness. Illicit drug use was also an exclusion criterion (only sporadic cannabis use was tolerated). Somatic comorbidities, including autoimmune diseases, or risk factors, such as a history of brain injury, led to exclusion from the study. The control participants were not allowed to take any psychopharmacological drugs, anticonvulsants, or immunomodulatory drugs. The screening approach has been described in detail elsewhere (44, 45, 46).

MRI-related exclusion criteria for the APS, BPD, and HC groups included pregnancy and lactation, lack of legal capacity, and inability to understand the scope of the study, as well as claustrophobia, pacemakers, metallic foreign bodies, or intrauterine contraceptive devices.

The psychometric test battery included questionnaires for BPD-associated symptoms, including the Borderline Symptom List-23 (BSL-23), the Difficulties in Emotion Regulation Scale, and the Dissociative Experiences Scale (FDS-20). To assess psychotic and affective symptoms, the Eppendorf Schizophrenia Inventory (ESI) and the Beck Depression Inventory–II (BDI-II) were collected. In addition, neuropsychological testing was performed including the Culture Fair Intelligence Testing (CFT-20-R) and the Multiple-Choice Vocabulary Test (MWT-B). The complete test battery was published in previous publications (44, 45, 46). Missing questionnaires did not lead to exclusion from the study, as some patients with APS were unable to complete the questionnaires due to their illness.

### EEG Measurement and Analysis

**Figure 2:**
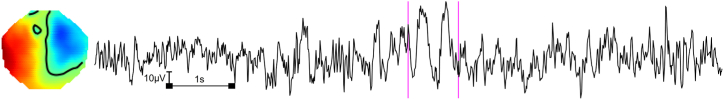
Representative example of an intermittent rhythmic theta activity (IRTA) detection in one of the participants with an autoimmune psychiatric syndrome included in the current study. Independent component analysis (ICA) component 14, start and end of the detection are marked by pink bars. A small residue of the gradient artifact can be seen. Left: topography of ICA component 14, viewed from the top; nose is at the top.

During the fMRI session, EEG was recorded using a 64-channel BrainAmp MR Plus system (Brain Products GmbH) at a sampling frequency of 5 kHz driven by the 10-MHz MR system clock signal via a SyncBox (Brain Products GmbH) to avoid sampling the high-frequency gradient artifact at slightly different time points. Offline, the averaged gradient artifact for each 100 ms TR was subtracted (53). Afterward, independent component analysis (ICA) (54) was used to detect components of ballistocardiographic (BCG) and eye movement artifacts. Within the ICA component traces not flagged as artifacts, an automated IRDA/IRTA detection procedure was applied (7) with a relatively lenient threshold of 4.5 μV. An example of a slight IRDA signal that would have been difficult to detect visually is shown in Figure 2. This threshold typically leads to few detections within the 10-minute fMRI scan, even in healthy individuals, allowing the inclusion of all subjects in the analysis of IRDA/IRTA-related fMRI activity.

### MRI Measurements

MRI scans were performed on a Magnetom Prisma 3T system (Siemens Healthineers). A 64-channel head coil was used for signal reception. Anatomical imaging was performed using a high-resolution T1-weighted magnetization-prepared rapid gradient-echo sequence (FOV = 256 × 256 × 160 mm^3^, voxel size = 1 × 1 × 1 mm^3^; TR = 2000 ms, TE = 4.11 ms). The MREG measurement was performed with a TE of 33 ms and a TR of 100 ms at a flip angle set to 21° (39,55,56). The scan time was 10 minutes (6000 whole-brain scans with 50 axial slices of 64 × 64 voxels, 3-mm isotropic resolution), with 50 dummy scans (5 seconds) eliminated at the start to allow for signal saturation. During this time, the subjects were instructed to keep their eyes fixated on a white cross displayed on a monitor behind the scanner bore and visible through a mirror mounted on the head coil. Respiration was recorded with a chest strap, and the heartbeat was recorded with a 4-channel electrocardiography (Siemens Healthineers).

### MRI Analysis and Statistics

First, image reconstruction and volume registration were carried out using custom MATLAB code, recording the 6 movement parameters per volume for later use. Further analysis was carried out using the AFNI software (57). The anatomical images were normalized using “sswarper2”; the AFNI meta-script “afni_proc.py” was used for despiking, alignment, registration on the normalized anatomy, and masking. The signals were further blurred with a 4-mm full width at half maximum Gaussian and normalized to percentage of signal change. Finally, in a regression analysis, the signals were corrected for the 6 movement parameters as well as 13 parameters for heartbeat and respiration phases and amplitudes extracted using “physio_calc.py” (58). The first-level analysis of IRDA/IRTA-related fMRI activity was performed using AFNI “3dDeconvolve,” with the detected IRDA/IRTA ranges being transformed into a regressor with a canonical hemodynamic response function via the AFNI “waver” program. In the second-level analysis (across subjects), functional correlations across all subjects (consensus areas) were analyzed first. The AFNI program “3dttest++” with the “-Clustsim” option performed voxelwise tests of difference from zero and derived voxel *p* thresholds and minimum cluster sizes *N* (in voxels) corresponding to a global alpha level of *p* < .05. Across all groups, the required voxel threshold was *p* < .003 and *n* > 43. Corresponding clusters were derived using “3dClusterize.” In the second step, the 3 groups were analyzed separately, setting *p* < .01 as the threshold and keeping *n* > 43 to achieve a similar level of sensitivity despite the smaller groups. For each group, clusters from this analysis that did not overlap with the consensus areas were noted. Further statistical analyses were performed using R version 3.6.0 (R Foundation for Statistical Computing Platform). Group comparisons for categorical variables (e.g., sex) were performed using a χ^2^ test. Continuous variables were analyzed using linear analysis of covariance, including age as a covariate of no interest. Rather than arbitrarily splitting groups into subsamples with and without IRDA/IRTA, the number of automatically detected IRDA/IRTA was used as a continuous variable. The “Brodmann_Pijn_AFNI” atlas was used to map the clusters to the respective Brodmann areas (BAs) (59). The anatomical and functional classification of the BAs was taken from the following home page: https://neurofeedback-academy.com/brodmann-area (accessed May 12, 2025). To investigate the associations between the IRDA/IRTA rates (measured within the scanner during the EEG-fMRI) and the detected functional clusters (which have been identified in EEG-fMRI as functional correlates of IRTA/IRTA activity) with clinical symptomatology, the following approach was applied: Linear models were calculated separately for each of the APS and BPD groups, with mean-corrected age (and in the APS group, also sex) as effects of no interest, with the respective psychometric scores as the dependent variable. As independent variables, 1) the IRDA/IRTA density within the scanner and 2) the mean activation or deactivation of the functional clusters were analyzed. Here, a significance level of *p* < .05 was used due to the exploratory approach.

## Results

### Patient Groups and HCs

Thirty-three patients with suspected APS (mean age: 40.8 ± 14.2 years; 52% female), 59 patients with BPD (mean age: 29.6 ± 8.9 years; 100% female), and 43 HC participants (mean age: 29.9 ± 10 years; 65% female) were analyzed. The score for BPD symptom severity, the BSL-23 score, was highest in the BPD group (*p* < .001; *n* = 129), and the ESI score for psychosis and its subscores were highest in the APS group (all subscores: *p* < .001; *n* = 125). The FDS-20 score for dissociation (*n* = 127) and BDI-II scores for depressive symptoms (*n* = 129) were highest in the BPD group, while the patients in the APS group also showed higher scores than the HC group (both *p* < .001). The neuropsychological test results also showed clear differences between the 3 groups, including CFT-20-R IQ with poorer performance in the patient groups (*p* < .001; *n* = 111). However, crystallized IQ as measured by the MWT-B showed no relevant differences between the 3 groups (*p* = .246; *n* = 127). At the time of the study, psychopharmacological medication was prescribed for 79% of the APS group (61% had additionally received immunotherapies), 85% of the BPD group, and 0% of the HC group. Further findings are summarized in Table 1. The detailed diagnostic findings (including, e.g., the associated antibody and CSF findings) of the APS group are summarized in Table S1.

**Table 1:** The Transdiagnostic Patient and Control Groups

	APS Group, *n* = 33	BPD Group, *n* = 59	Control Group, *n* = 43	Statistics *p* Value^a^
Age, Years	40.8 ± 14.2	29.6 ± 8.9	29.9 ± 10.0	<.001∗
Sex				
Female	17 (52%)	59 (100%)	28 (65%)	<.001∗
Male	16 (48%)	0 (0%)	15 (35%)	
Handedness				
Right	23 (82%) (*n* = 28)	50 (89%) (*n* = 56)	37 (88%) (*n* = 42)	.607
Left	2 (7%) (*n* = 28)	4 (7%) (*n* = 56)	4 (10%) (*n* = 42)	
Both	3 (11%) (*n* = 28)	2 (4%) (*n* = 56)	1 (2%) (*n* = 42)	
Mother Tongue				
German	32 (97%)	54 (96%) (*n* = 56)	34 (81%) (*n* = 42)	.011∗
English	0 (0%)	0 (0%) (*n* = 56)	2 (5%) (*n* = 42)	
French	0 (0%)	0 (0%) (*n* = 56)	3 (7%) (*n* = 42)	
Romanian	1 (3%)	0 (0%) (*n* = 56)	2 (5%) (*n* = 42)	
Chinese	0 (0%)	0 (0%) (*n* = 56)	1 (2%) (*n* = 42)	
Russian	0 (0%)	2 (4%) (*n* = 56)	0 (0%) (*n* = 42)	
Body Mass Index	25.7 ± 5.3	25.9 ± 6.3 (*n* = 55)	22.0 ± 2.3 (*n* = 42)	<.001∗
Academic Degree				
University degree	8 (24%)	6 (11%) (*n* = 56)	23 (55%) (*n* = 42)	<.001∗
High degree	9 (27%)	22 (39%) (*n* = 56)	17 (40%) (*n* = 42)	
Middle degree	10 (30%)	25 (45%) (*n* = 56)	2 (5%) (*n* = 42)	
Low degree	4 (12%)	3 (5%) (*n* = 56)	0 (0%) (*n* = 42)	
Other qualification	2 (6%)	0 (0%) (*n* = 56)	0 (0%) (*n* = 42)	
Current Employment Status				
Full-time job	2 (6%)	3 (5%) (*n* = 56)	13 (31%) (*n* = 42)	<.001∗
Part-time job	7 (21%)	18 (32%) (*n* = 56)	6 (31%) (*n* = 42)	
Student	4 (12%)	9 (16%) (*n* = 56)	21 (50%) (*n* = 42)	
Trainee	1 (3%)	2 (4%) (*n* = 56)	1 (2%) (*n* = 42)	
Pensioner	12 (36%)	4 (7%) (*n* = 56)	0 (0%) (*n* = 42)	
Unemployed	7 (21%)	20 (36%) (*n* = 56)	1 (2%) (*n* = 42)	

Values are presented as mean ± SD or *n* (%). Not all data were available in full, so the number of patients/control participants for whom data were available is shown in brackets.

∗Significant difference.

APS, autoimmune psychiatric syndromes; BPD, borderline personality disorder.

a Kruskal-Wallis rank-sum test, Fisher’s exact test, or Pearson’s χ^*2*^ test.

### IRDA/IRTA Rates in Simultaneous EEG-fMRI Measurements

**Figure 3:**
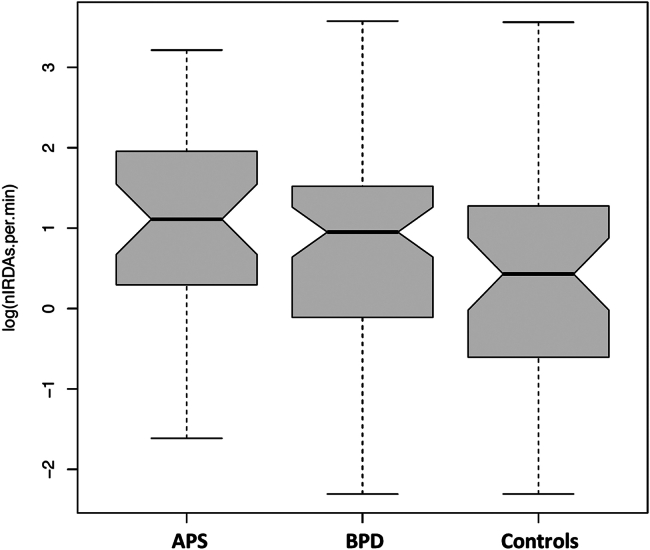
Boxplots of intermittent rhythmic delta/theta activity from the electroencephalography (EEG) detected during combined EEG and functional magnetic resonance imaging (MRI) measurements within the MRI scanner. The notch ranges around each median approximate the 95% CI. APS, autoimmune psychiatric syndromes; BPD, borderline personality disorder.

The IRDA/IRTA rates (without hyperventilation) within the MRI scanner were highest in the APS group. The BPD group also had higher IRDA/IRTA rates than the control group (see Figure 3).

### Functional Correlates of IRDA/IRTA

**Figure 4:**
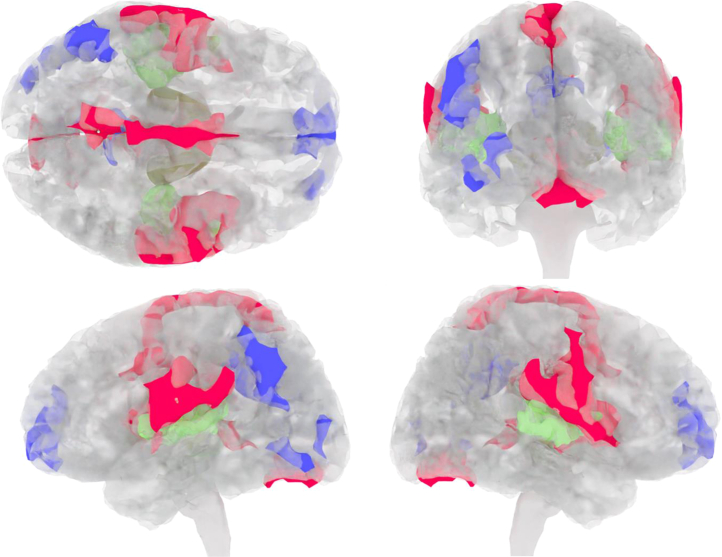
Correlates of intermittent rhythmic delta/theta activity and activation in functional magnetic resonance imaging (fMRI) (marked in red: increased activation, marked in blue: reduced activation, marked in green: fMRI activation in a simple auditory block paradigm for anatomical orientation). Top, back, left, and right view are shown.

**Figure 5:**
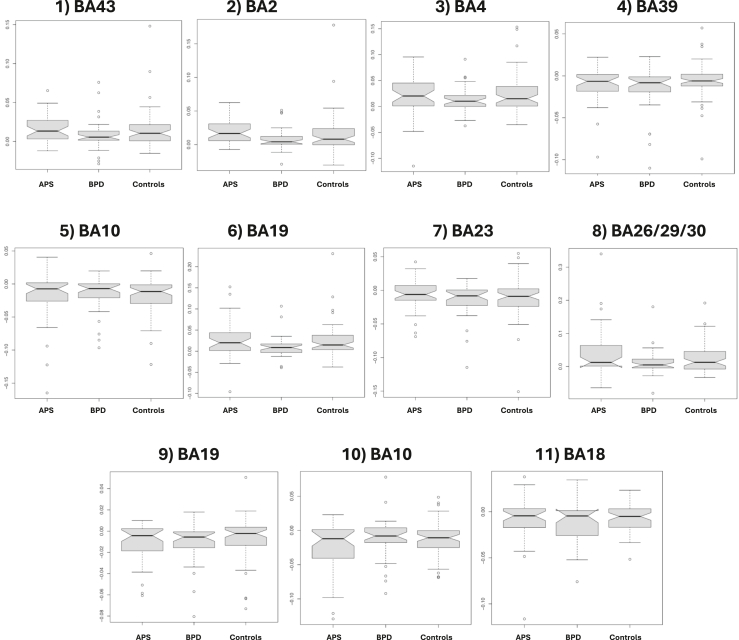
Clusters 1 to 11 (see Table 3) are functional consensus areas that were significant across the 3 groups, i.e., in the whole cohort. APS, autoimmune psychiatric syndromes; BA, Brodmann area; BPD, borderline personality disorder.

**Figure 6:**
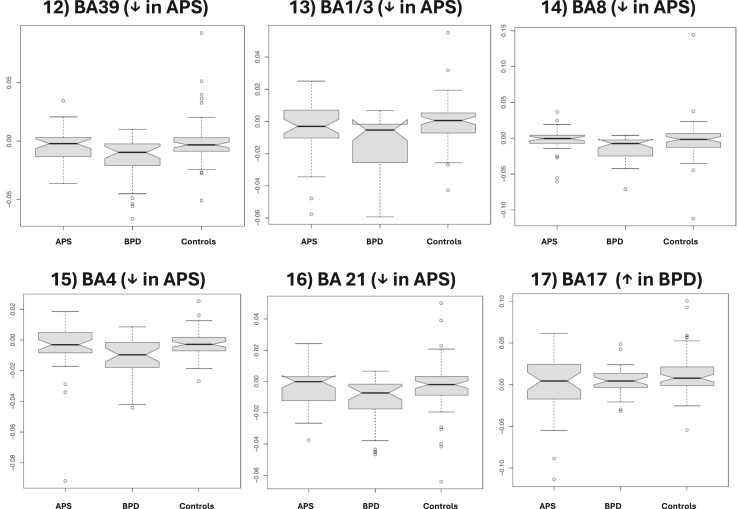
The groups were analyzed individually and significant clusters that did not overlap with 1 to 11 (Figure 4) were added. Clusters 12 to 16 are those added in the autoimmune psychiatric syndrome (APS) group (all negative: ↓), and cluster 17 (positive: ↑) was added in the borderline personality disorder (BPD) group (see Table 3). BA, Brodmann area.

In all 3 groups, altered brain activity correlating with IRDA/IRTA in fMRI was detected in several consensus areas. The findings are summarized in Table 2 and Figures 4 and 5. Eleven consensus areas that were significant across the 3 groups were identified: cluster 1 in BA 43-right (r), cluster 2 in BA 2-left (l), cluster 3 in BA 4-r, cluster 4 in BA 39-l, cluster 5 in BA 10-l, cluster 6 in BA 18-r, cluster 7 in BA 23-l, cluster 8 in BA 26/29/30-r, cluster 9 in BA 19-l, cluster 10 in BA 10-r, and cluster 11 in BA 18-l. The order reflects the descending volumes of the consensus areas, i.e., BA 43-r contained the largest correlating functional area, and BA 18-l contained the smallest. Five consensus generator areas showed increased activity (marked in red in Figure 4): BA 43-r (superior middle temporal lobe), BA 2-l (middle postcentral gyrus), BA 4-r (posterior frontal lobe), BA 18-r (lateral occipital lobe), and BA 26/29/30-r (cingulate cortex). Six areas had reduced activity (marked in blue in Figure 4): BA 39-l (lateral parietal lobe), BA 10-l (anterior prefrontal cortex), BA 23-l (posterior cingulate), BA 19-l (dorsolateral occipital lobe), BA 10-r (anterior prefrontal cortex), and BA 18-l (lateral occipital lobe). There were no differences across the 3 groups in the number of voxels involved in these regions (Figure 5). The groups were then analyzed individually, and significant clusters were added that did not overlap with the 11 consensus clusters. Five additional clusters (clusters 12–16) were identified in the APS group (all with reduced activity). The areas were located in BA 39-r (lateral parietal lobe; cluster 12), BA 1/3-r (middle postcentral gyrus; cluster 13), BA 8-r (frontal cortex; cluster 14), BA 4-l (posterior frontal lobe; cluster 15), and BA 21-r (middle temporal lobe; cluster 16). The order also reflects descending volumes, i.e., BA 39-r shows the largest area with reduced activity in the APS group. One further cluster was detected in BA 17-l (posterior occipital lobe; cluster 17) with increased activity in the BPD group. The statistical differences between these additional areas are summarized in Figure 6. No further clusters were detected for the HC group.

**Table 2:** Description of the 17 Detected Clusters Including BA and Centroid Coordinates

Area	Cluster	BA	Activation	Hemisphere	Volume, mm^3^	CM_RL	CM_AP	CM_IS	minRL	maxRL	minAP	maxAP	minIS	maxIS
Consensus	1	BA 43	↑	Right	990	−51.6	6.6	18	−67.5	−31.5	−19.5	34.5	−10.5	58.5
Consensus	2	BA 2	↑	Left	734	52.9	11.6	19.4	34.5	67.5	−16.5	43.5	−10.5	43.5
Consensus	3	BA 4	↑	Right	391	2.2	21.7	60.9	−10.5	16.5	−19.5	73.5	31.5	79.5
Consensus	4	BA 39	↓	Left	263	49.6	60.7	35.9	40.5	61.5	46.5	73.5	13.5	61.5
Consensus	5	BA 10	↓	Left	255	2.7	−59.9	3.4	−13.5	16.5	−70.5	−49.5	−19.5	28.5
Consensus	6	BA 18	↑	Right	220	−5.5	78.2	−19.9	−28.5	10.5	61.5	91.5	−28.5	−10.5
Consensus	7	BA 23	↓	Left	138	4.2	51.1	33.6	−16.5	13.5	37.5	70.5	19.5	46.5
Consensus	8	BA 26/29/30	↑	Right	90	−1.5	40.6	1.7	−13.5	10.5	31.5	55.5	−7.5	13.5
Consensus	9	BA 19	↓	Left	68	42.9	76.6	−10.9	34.5	49.5	61.5	88.5	−19.5	−1.5
Consensus	10	BA 10	↓	Right	50	−27.1	−59	5	−34.5	−19.5	−64.5	−52.5	−1.5	10.5
Consensus	11	BA 18	↓	Left	46	30.2	92.7	1.5	22.5	37.5	88.5	97.5	−13.5	10.5
From APS	12	BA 39	↓	Right	415	−47.1	59	35.4	−64.5	−25.5	37.5	82.5	19.5	52.5
From APS	13	BA 1/3	↓	Right	129	−41	20.4	51.2	−49.5	−31.5	10.5	31.5	37.5	64.5
From APS	14	BA 8	↓	Right	114	−16.2	−32.1	43	−34.5	10.5	−43.5	−13.5	34.5	49.5
From APS	15	BA 4	↓	Left	82	32.9	21.8	56.9	13.5	43.5	13.5	31.5	37.5	70.5
From APS	16	BA 21	↓	Right	63	−61.2	38.4	−15.4	−67.5	−55.5	28.5	46.5	−25.5	−4.5
From BPD	17	BA 17	↑	Left	121	1.9	80.8	10.6	−10.5	13.5	67.5	94.5	−4.5	28.5

Clusters 1 to 11 are consensus areas that were significant across the 3 groups. The groups were then analyzed individually, and significant clusters that did not overlap with 1 to 11 were added. Clusters 12 to 16 are those added in the APS group (all negative), and cluster 17 (positive) is added in the BPD group. The order of the clusters follows the volume (starting with the largest volume and then descending). No clusters were added for the control group. Some BAs appear 2 times (e.g., BA 39). In this case, the functional correlations were located in different hemispheres.

AP, anterior-posterior; APS, autoimmune psychiatric syndromes; BA, Brodmann area; BPD, borderline personality disorder; CM, center of mass; IS, inferior-superior; max, maximum; min, minimum; RL, right-left; ROI, region of interest.

### Association of Psychometrics With IRDA/IRTA and Functional Clusters

IRDA/IRTA rates (measured within the scanner) correlated with the ESI subscore for attention and speech impairment (ESI-AS) in the APS group (coefficient [C] = 0.51; *p* = .019) and with the ESI subscore frankness impairment (ESI-FR) in the BPD group (C = 0.21; *p* = .005). Both were positive associations (i.e., higher IRDA/IRTA rates were related to more symptoms), indicated by the positive coefficients. For the 17 functional clusters detected in EEG-fMRI analyses, there were 4 significant relationships in total, 1 with reduced activity for APS (BA 21-r = cluster 16, additional in the APS group, and ESI deviant perception impairment: C = −134.58; *p* = .043) and 3 for BPD (BA 26/29/30-r = cluster 8, consensus area, and ESI subscore ESI-AS: C = −42.78; *p* = .041; BA 26/29/30-r = cluster 8, consensus area, and BSL-23: C = −5.04; *p* = .045; BA 19-l = cluster 9, consensus area, and ESI subscore ESI-FR: C = 51.36; *p* = .025) (Table 3).

**Table 3:** Association Between Psychometric Scores and IRDA/IRTA Within the Scanner and the 17 Detected Functional Clusters (Mean Functional Activation, See Table 2)

		IRDA/IRTA, Within the Scanner, Rate per min		Functional Cluster 1, Mean Activation		Functional Cluster 2, Mean Activation		Functional Cluster 3, Mean Activation		Functional Cluster 4, Mean Activation		Functional Cluster 5, Mean Activation	
Group	Measure	C	*p*	C	*p*	C	*p*	C	*p*	C	*p*	C	*p*
APS	ESI-AS	0.51	.019	44.37	.519	62.31	.505	−20.15	.732	−29.71	.624	8.22	.871
	ESI-AU	0.15	.500	103.94	.112	122.19	.172	43.26	.445	23.12	.682	10.84	.818
	ESI-DP	−0.02	.907	85.77	.120	84.39	.265	33.94	.477	−2.09	.965	−4.69	.905
	ESI-IR	0.14	.499	100.54	.113	109.51	.208	42.70	.441	15.76	.776	−1.16	.980
	ESI-FR	0.06	.615	17.64	.619	2.81	.954	8.47	.780	19.44	.518	13.04	.597
	BDI-II	0.52	.343	−84.84	.601	−107.20	.582	−33.16	.809	23.67	.826	143.94	.221
	BSL-23	0.02	.471	−4.99	.527	−5.49	.562	0.57	.932	−0.37	.944	2.24	.700
BPD	ESI-AS	0.02	.883	−14.97	.680	−16.05	.601	−26.63	.281	1.64	.970	−2.46	.941
	ESI-AU	0.03	.744	−3.26	.899	−1.08	.960	−0.53	.976	56.73	.062	15.98	.502
	ESI-DP	0.06	.605	−13.75	.626	−9.22	.700	−13.88	.472	36.39	.287	19.72	.449
	ESI-IR	0.03	.759	−9.00	.685	−4.61	.807	1.01	.947	34.30	.194	11.41	.578
	ESI-FR	0.21	.005	−5.41	.780	−14.50	.376	−8.52	.520	−7.87	.735	−4.26	.812
	BDI-II	0.40	.109	−12.38	.849	−31.49	.567	−25.16	.570	−85.95	.267	−34.93	.559
	BSL-23	0.03	.116	−4.82	.267	−4.44	.227	−3.80	.199	−5.48	.292	−2.58	.520

		Functional Cluster 6, Mean Activation		Functional Cluster 7, Mean Activation		Functional Cluster 8, Mean Activation		Functional Cluster 9, Mean Activation		Functional Cluster 10, Mean Activation		Functional Cluster 11, Mean Activation	
Group	Measure	C	*p*	C	*p*	C	*p*	C	*p*	C	*p*	C	*p*
APS	ESI-AS	2.77	.951	−6.21	.925	−17.60	.695	75.49	.404	1.35	.977	52.84	.476
	ESI-AU	10.47	.811	23.99	.698	−6.15	.888	68.90	.431	14.78	.748	49.80	.485
	ESI-DP	17.12	.641	−7.74	.882	−12.52	.733	26.05	.724	14.91	.697	34.82	.562
	ESI-IR	0.63	.988	18.82	.757	−12.97	.760	53.01	.532	−0.70	.987	41.40	.557
	ESI-FR	3.11	.894	38.27	.246	−3.07	.894	−3.36	.942	14.08	.559	44.65	.232
	BDI-II	15.43	.884	77.55	.611	−66.15	.353	77.30	.626	90.07	.421	79.25	.629
	BSL-23	3.67	.474	0.90	.904	−1.26	.718	−0.47	.951	2.36	.666	2.46	.759
BPD	ESI-AS	−24.71	.272	−18.87	.532	−42.78	.041	−25.44	.561	34.90	.326	−18.03	.758
	ESI-AU	−0.81	.960	15.80	.462	−16.91	.262	48.27	.118	8.52	.737	56.03	.175
	ESI-DP	−11.20	.525	4.91	.835	−21.90	.207	29.52	.386	15.49	.579	61.41	.175
	ESI-IR	0.76	.956	8.83	.634	−9.44	.469	28.35	.289	18.21	.404	62.13	.079
	ESI-FR	−12.82	.287	−8.92	.581	−16.59	.142	51.36	.025	12.89	.499	52.67	.088
	BDI-II	−8.63	.831	−36.03	.505	−40.83	.281	58.53	.455	28.33	.656	−69.22	.509
	BSL-23	−4.26	.113	−4.43	.220	−5.04	.045	2.77	.599	−0.16	.971	−9.10	.193
		Functional Cluster 12, Mean Activation		Functional Cluster 13, Mean Activation		Functional Cluster 14, Mean Activation		Functional Cluster 15, Mean Activation		Functional Cluster 16, Mean Activation		Functional Cluster 17, Mean Activation	

Group	Measure	C	*p*	C	*p*	C	*p*	C	*p*	C	*p*	C	*p*

APS	ESI-AS	67.55	.368	65.20	.348	−39.01	.676	120.95	.208	−80.82	.348	55.90	.511
	ESI-AU	90.66	.189	66.50	.326	−43.86	.622	66.45	.475	−98.84	.223	95.31	.246
	ESI-DP	56.58	.334	38.77	.491	−78.45	.287	7.19	.927	−134.58	.043	60.88	.378
	ESI-IR	88.83	.190	60.88	.350	−35.00	.688	80.33	.376	−90.41	.250	77.89	.335
	ESI-FR	57.20	.120	33.30	.346	27.22	.573	55.70	.257	−19.64	.656	41.08	.349
	BDI-II	129.74	.399	150.67	.314	142.01	.418	182.80	.383	−28.65	.883	−42.72	.819
	BSL-23	−0.26	.973	0.09	.991	1.53	.859	3.03	.768	−1.59	.867	6.95	.441
BPD	ESI-AS	−36.16	.416	−55.16	.388	−37.91	.239	−62.60	.570	00.29	.996	−19.52	.570
	ESI-AU	21.61	.495	16.38	.720	11.06	.631	32.87	.675	43.83	.244	−10.89	.656
	ESI-DP	−1.96	.955	20.65	.679	1.52	.953	30.16	.726	22.46	.588	−16.54	.537
	ESI-IR	5.23	.848	12.94	.742	3.28	.869	33.05	.624	9.52	.770	−1.28	.952
	ESI-FR	−29.64	.211	−10.52	.759	−20.16	.242	79.33	.175	−15.18	.594	13.97	.447
	BDI-II	−71.73	.368	−184.51	.104	−46.82	.418	−116.06	.544	−58.76	.537	63.72	.298
	BSL-23	−8.97	.090	−11.52	.131	−5.69	.139	−2.49	.847	−3.45	.589	−0.73	.861

APS, autoimmune psychiatric syndromes; AS, attention and speech impairment; AU, auditory uncertainty impairment; BDI, Beck Depression Inventory; BPD, borderline personality disorder; BSL, Borderline Symptom List; C, coefficient; DP, deviant perception impairment; ESI, Eppendorf Schizophrenia Inventory; FR, frankness impairment; IR, ideas of reference impairment; IRDA, intermittent rhythmic delta activity; IRTA, intermittent rhythmic theta activity.

## Discussion

The main result of this study is the detection of a network of transdiagnostic functional consensus areas associated with IRDA/IRTA events. This network comprised consensus areas with increased activity (including frontal and temporal areas) and regions of reduced activity, e.g., in the prefrontal cortex. Additional non-overlapping, disease-specific areas with reduced activity were identified in the APS group, and one further disorder-specific occipital region with increased activity was found in the BPD group.

The consensus areas, which may represent transdiagnostic functional correlates of IRDA/IRTA, included 11 BAs, 5 of them with increased activity in the following locations/regions: BA 43 (superior middle temporal lobe: primary gustatory cortex, Wernicke’s area), BA 2 (middle postcentral gyrus: primary somatosensory cortex), BA 4 (posterior frontal lobe: primary motor cortex), BA 18 (lateral occipital lobe: secondary visual cortex), and BA 26/29/30 (isthmus of cingulate gyrus/posterior cingulate: parts of the cingulate cortex). Temporal, postcentral, frontal, and parts of the cingulum seem to be particularly affected. Similar findings with temporal and frontal functional generators were observed in epilepsies in EEG-fMRI (35,37). In addition, patterns with comparable increased activity were identified in the context of anticipating painful brain stimuli (60). The similarity with the higher-level salience network could be consistent with the fact that a higher integration center is disturbed by IRDA/IRTA events (61). The functionally activated regions could indicate that IRDA/IRTA directly or indirectly represent an excitatory electrophysiological activity (comparable with epileptic activity). In contrast, the following 6 areas showed reduced activity: BA 39 (lateral parietal lobe: angular gyrus), BA 10 (anterior prefrontal cortex: parts of the prefrontal cortex), BA 23 (posterior cingulate: ventral posterior cingulate cortex), BA 19 (dorsolateral occipital lobe: associate visual cortex), BA 10B (anterior prefrontal cortex: parts of the prefrontal cortex), and BA 18 (lateral occipital lobe: secondary visual cortex). According to the LANI hypothesis, the relatively global hypoactivity could represent a compensatory, counterregulatory consequence of excitatory IRDA/IRTA events. It may cause network-dependent symptoms when it exceeds a certain threshold (28). Our correlative analyses revealed an association between high IRDA/IRTA rates within the scanner and attention plus speech impairment in the APS group and with frankness impairment in the BPD group; accordingly, the IRDA/IRTA phenomena could potentially have some clinical relevance.

Additionally, disease- or disorder-specific functional clusters were identified. Five non-overlapping clusters, all with reduced activity, were detected in the APS group. These clusters were located in BA 39 (lateral parietal lobe: angular gyrus), BA 1/3 (middle postcentral gyrus: primary somatosensory cortex), BA 8 (frontal cortex: frontal eye fields), BA 4 (posterior frontal lobe: primary motor cortex), and BA 21 (middle temporal lobe: middle temporal gyrus). The functional deactivation in BA 21 (middle temporal lobe: middle temporal gyrus), which was also detected in the APS group, correlated with symptoms of deviant perception impairment in this patient group and therefore could be associated in particular with the clinical symptoms in APS resulting from high IRDA/IRTA density. If clinical symptoms are interpreted as consequences of a compensatory LANI (caused by excitatory IRDA/IRTA) (28), then this additional global pattern of changes would fit with the fact that the symptoms of APS are characterized by broad psychopathological changes (16,40,41,62). The characteristic feature of these APS cases is usually diverse and thus often atypical (16,18,40,41,62). One further cluster with increased activity in BA 17 (posterior occipital lobe: primary visual cortex) was identified in the BPD group. Consistent with this, recent MRI studies showed a link between emotion processing and the connectivities of the visual cortex. Hyperresponsiveness of the visual cortex has been observed in particular in the processing of emotional stimuli such as viewing emotional faces (63).

Conceptually, the findings could be integrated into the LANI model (28), which postulates that IRDA/IRTA represents excitatory EEG activity, shown here in the form of a network with 5 consensus clusters with increased activity. According to the LANI hypothesis, ongoing psychological symptoms could be the consequence of secondary compensatory local network inhibition. This would be consistent with the fact that 6 consensus clusters with reduced activity were detected across the groups. The areas with reduced activity were relatively globally distributed, which could potentially explain different resulting symptoms. It should also be noted that additional non-overlapping regions of altered activity were found in the 2 patient groups. The relatively global picture with non-overlapping reduced activity in APS is consistent with the previously described global hypoconnectivities in patients with autoimmune encephalitis (64, 65, 66), the relatively global brain-binding of some CNS antibodies (48), and the associated frequently complex clinical pictures of APS (18,40,41,62). Specific aspects involving the increased activity of the visual system match the emotional symptomatology in BPD (43,63). Additional non-overlapping clusters were not observed in the HC group (in contrast to the patient groups), supporting the disease- respectively disorder-specific nature of the findings in both patient groups. However, correlational analyses of the functional clusters with the psychometric scores only partially confirmed these associations.

In terms of limitations, it is possible that the results might have been influenced by other causal or different modulating factors. The transdiagnostic approach took into account the typical populations for the respective disease/disorder, resulting in age and sex differences that could have influenced the results. However, the aim of this study was not to investigate the underlying causalities but rather to understand the pathophysiology of the IRDA/IRTA phenomenon itself. The transdiagnostic approach allowed us to compare patients with a suspected uniform autoimmune-mediated causality, patients with a clearly defined primary psychiatric etiology in a personality disorder, and HC participants. The interpretation of the results should not be overstated, as we can only show a correlation between IRDA/IRTA and functional BOLD activity but cannot make detailed statements about related causalities. The EEG noise level within the MRI scanner is increased with respect to well-shielded EEG laboratories despite state-of-the art reduction of gradient and BCG (i.e., heartbeat-related) artifacts. Since movements in the scanner lead to removal of both EEG (due to large artifacts) and fMRI (censoring) data, we do not expect this to have systematic effects on our analyses. In summary, the findings provide a basis for further studies in the field, which should validate our results.

### Conclusions

This study identified transdiagnostic functional correlates of slow excitatory EEG activity, and the results were interpreted within the previously postulated LANI hypothesis. Further studies on non-epileptic excitatory EEG activity seem promising and should also investigate the effects of psychopharmacological interventions on the EEG phenomena in relation to clinical improvement as well as basic experimental research on IRDA/IRTA.

## Supplementary Material

Table S1

Key Resources Table

## References

[1] Walser H., Isler H. (1982). Frontal intermittent rhythmic delta activity. Impairment of consciousness and migraine. Headache.

[2] Matsuura M., Yoshino M., Ohta K., Onda H., Nakajima K., Kojima T. (1994). Clinical significance of diffuse delta EEG activity in chronic schizophrenia. Clin Electroencephalogr.

[3] Neufeld M.Y., Chistik V., Chapman J., Korczyn A.D. (1999). Intermittent rhythmic delta activity (IRDA) morphology cannot distinguish between focal and diffuse brain disturbances. J Neurol Sci.

[4] Calzetti S., Bortone E., Negrotti A., Zinno L., Mancia D. (2002). Frontal intermittent rhythmic delta activity (FIRDA) in patients with dementia with Lewy bodies: A diagnostic tool?. Neurol Sci.

[5] Watemberg N., Alehan F., Dabby R., Lerman-Sagie T., Pavot P., Towne A. (2002). Clinical and radiologic correlates of frontal intermittent rhythmic delta activity. J Clin Neurophysiol.

[6] Tebartz van Elst L., Fleck M., Bartels S., Altenmüller D.M., Riedel A., Bubl E. (2016). Increased prevalence of intermittent rhythmic delta or theta activity (IRDA/IRTA) in the electroencephalograms (EEGs) of patients with borderline personality disorder. Front Behav Neurosci.

[7] Endres D., Maier S., Feige B., Mokhtar N.B., Nickel K., Goll P. (2017). Increased rates of intermittent rhythmic delta and theta activity in the electroencephalographies of adult patients with attention-deficit hyperactivity disorder. Epilepsy Behav.

[8] Endres D., Maier S., Feige B., Posielski N.A., Nickel K., Ebert D. (2017). Altered intermittent rhythmic delta and theta activity in the electroencephalographies of high functioning adult patients with autism spectrum disorder. Front Hum Neurosci.

[9] Cerrahoğlu Şirin T., Bekdik Şirinocak P., Arkalı B.N., Akıncı T., Yeni S.N. (2019). Electroencephalographic features associated with intermittent rhythmic delta activity. Neurophysiol Clin.

[10] Endres D., Reinhold E., Klesse C., Domschke K., Prüss H., Tebartz van Elst L. (2025). Suspected autoimmune-mediated dissociative symptoms. Mol Psychiatry.

[11] Endres D., Perlov E., Feige B., Fleck M., Bartels S., Altenmüller D.M., Tebartz van Elst L. (2016). Electroencephalographic findings in schizophreniform and affective disorders. Int J Psychiatry Clin Pract.

[12] Graus F., Titulaer M.J., Balu R., Benseler S., Bien C.G., Cellucci T. (2016). A clinical approach to diagnosis of autoimmune encephalitis. Lancet Neurol.

[13] Dalmau J., Graus F. (2018). Antibody-mediated encephalitis. N Engl J Med.

[14] Titulaer M.J., McCracken L., Gabilondo I., Armangué T., Glaser C., Iizuka T. (2013). Treatment and prognostic factors for long-term outcome in patients with anti-NMDA receptor encephalitis: An observational cohort study. Lancet Neurol.

[15] Gillinder L., Warren N., Hartel G., Dionisio S., O’Gorman C. (2019). EEG findings in NMDA encephalitis—A systematic review. Seizure.

[16] Pollak T.A., Lennox B.R., Müller S., Benros M.E., Prüss H., Tebartz van Elst L. (2020). Autoimmune psychosis: An international consensus on an approach to the diagnosis and management of psychosis of suspected autoimmune origin. Lancet Psychiatry.

[17] Endres D., Leypoldt F., Bechter K., Hasan A., Steiner J., Domschke K. (2020). Autoimmune encephalitis as a differential diagnosis of schizophreniform psychosis: Clinical symptomatology, pathophysiology, diagnostic approach, and therapeutic considerations. Eur Arch Psychiatry Clin Neurosci.

[18] Tebartz van Elst L., Runge K., Meyer P.T., Urbach H., Venhoff N., Prüss H. (2025). The Neuropsychiatric Checklist for Autoimmune Psychosis: A narrative review. Biol Psychiatry.

[19] De la Fuente J.M., Bobes J., Vizuete C., Mendlewicz J. (2001). Sleep-EEG in borderline patients without concomitant major depression: A comparison with major depressives and normal control subjects. Psychiatry Res.

[20] De la Fuente J.M., Tugendhaft P., Mavroudakis N. (1998). Electroencephalographic abnormalities in borderline personality disorder. Psychiatry Res.

[21] Russ M.J., Campbell S.S., Kakuma T., Harrison K., Zanine E. (1999). EEG theta activity and pain insensitivity in self-injurious borderline patients. Psychiatry Res.

[22] Battaglia M., Ferini Strambi L., Bertella S., Bajo S., Bellodi L. (1999). First-cycle REM density in never-depressed subjects with borderline personality disorder. Biol Psychiatry.

[23] Reeves R.R., Struve F.A., Patrick G. (2003). EEG does not predict response to valproate treatment of aggression in patients with borderline and antisocial personality disorders. Clin Electroencephalogr.

[24] Boutros N.N., Torello M., McGlashan T.H. (2003). Electrophysiological aberrations in borderline personality disorder: State of the evidence. J Neuropsychiatry Clin Neurosci.

[25] Mina Y., Fahoum F., Abramovici S., Anis S., Kipervasser S. (2019). Clinical correlates and electroencephalographic features of FIRDA in a tertiary center. Acta Neurol Scand.

[26] Marcinski Nascimento K.J., Yuan D., Greenblatt A.S., Beniczky S., Nascimento F.A. (2025). Teaching NeuroImage: Temporal intermittent rhythmic delta activity: An epileptiform equivalent. Neurology.

[27] Tebartz van Elst L., Perlov E. (2013).

[28] Tebartz van Elst L., Krishnamoorthy E.S., Schulze-Bonhage A., Altenmüller D.M., Richter H., Ebert D., Feige B. (2011). Local area network inhibition: A model of a potentially important paraepileptic pathomechanism in neuropsychiatric disorders. Epilepsy Behav.

[29] Goldman R.I., Stern J.M., Engel J., Cohen M.S. (2002). Simultaneous EEG and fMRI of the alpha rhythm. NeuroReport.

[30] Jacobs J., Levan P., Moeller F., Boor R., Stephani U., Gotman J., Siniatchkin M. (2009). Hemodynamic changes preceding the interictal EEG spike in patients with focal epilepsy investigated using simultaneous EEG-fMRI. Neuroimage.

[31] Duncan J.S., Winston G.P., Koepp M.J., Ourselin S. (2016). Brain imaging in the assessment for epilepsy surgery. Lancet Neurol.

[32] Chaudhary U.J., Centeno M., Thornton R.C., Rodionov R., Vulliemoz S., McEvoy A.W. (2016). Mapping human preictal and ictal haemodynamic networks using simultaneous intracranial EEG-fMRI. NeuroImage Clin.

[33] Feige B., Spiegelhalder K., Kiemen A., Bosch O.G., Tebartz van Elst L., Hennig J. (2017). Distinctive time-lagged resting-state networks revealed by simultaneous EEG-fMRI. Neuroimage.

[34] Yoganathan K., Malek N., Torzillo E., Paranathala M., Greene J. (2023). Neurological update: Structural and functional imaging in epilepsy surgery. J Neurol.

[35] Wilson W., Tehrani N., Pittman D.J., Dykens P., Mosher V., Gill L. (2024). Mapping interictal discharges using intracranial EEG-fMRI to predict postsurgical outcomes. Brain.

[36] Ikemoto S., von Ellenrieder N., Gotman J. (2025). Interictal epileptiform discharge-related BOLD responses in the default mode network and subcortical regions. Clin Neurophysiol.

[37] Lin P.T., Sie J.H., Lee H.J., Chou C.C., Shih Y.C., Chen C. (2025). Detection of epileptogenic zones in people with epilepsy using optimized EEG-fMRI. Epilepsy Behav.

[38] Mäkiranta M.J., Ruohonen J., Suominen K., Sonkajärvi E., Salomäki T., Kiviniemi V. (2004). BOLD-contrast functional MRI signal changes related to intermittent rhythmic delta activity in EEG during voluntary hyperventilation-simultaneous EEG and fMRI study. Neuroimage.

[39] Hennig J., Kiviniemi V., Riemenschneider B., Barghoorn A., Akin B., Wang F., LeVan P. (2021). 15 years MR-encephalography. Magma.

[40] Endres D., Lüngen E., Hasan A., Kluge M., Fröhlich S., Lewerenz J. (2022). Clinical manifestations and immunomodulatory treatment experiences in psychiatric patients with suspected autoimmune encephalitis: A case series of 91 patients from Germany. Mol Psychiatry.

[41] Endres D., Maier V., Leypoldt F., Wandinger K.P., Lennox B., Pollak T.A. (2022). Autoantibody-associated psychiatric syndromes: A systematic literature review resulting in 145 cases. Psychol Med.

[42] Endres D., Meixensberger S., Dersch R., Feige B., Stich O., Venhoff N. (2020). Cerebrospinal fluid, antineuronal autoantibody, EEG, and MRI findings from 992 patients with schizophreniform and affective psychosis. Transl Psychiatry.

[43] Bohus M., Stoffers-Winterling J., Sharp C., Krause-Utz A., Schmahl C., Lieb K. (2021). Borderline personality disorder. Lancet.

[44] Endres D., Matteit I., von Zedtwitz K., Feige B., Schlump A., Reisert M. (2025). MR spectroscopic imaging and its association with EEG, CSF, and psychometric/neuropsychological findings in patients with suspected autoimmune psychosis spectrum syndromes. Acta Neuropsychiatr.

[45] Schlump A., Feige B., Matthies S., von Zedtwitz K., Matteit I., Lange T. (2025). Brain structural correlates of EEG network hyperexcitability, symptom severity, attention, and memory in borderline personality disorder. Brain Sci.

[46] von Zedtwitz K., Tebartz van Elst L., Feige B., Matteit I., Schlump A., Lange T. (2025). Morphometric MRI findings in patients with suspected autoimmune psychosis spectrum syndromes and association with EEG slowing, CSF changes, and psychometric/neuropsychological findings. Front Immunol.

[47] Kreye J., Reincke S.M., Kornau H.C., Sánchez-Sendin E., Corman V.M., Liu H. (2020). A therapeutic non-self-reactive SARS-CoV-2 antibody protects from lung pathology in a COVID-19 hamster model. Cell.

[48] Endres D., von Zedtwitz K., Matteit I., Bünger I., Foverskov-Rasmussen H., Runge K. (2022). Spectrum of novel anti-central nervous system autoantibodies in the cerebrospinal fluid of 119 patients with schizophreniform and affective disorders. Biol Psychiatry.

[49] Legge A.C., Hanly J.G. (2024). Recent advances in the diagnosis and management of neuropsychiatric lupus. Nat Rev Rheumatol.

[50] Endres D., von Zedtwitz K., Nickel K., Runge K., Maier A., Domschke K. (2024). Association of rheumatological markers with neuronal antibodies, cerebrospinal fluid, electroencephalography, and magnetic resonance imaging findings in 224 patients with psychotic syndromes. Brain Behav Immun.

[51] Al-Diwani A., Pollak T.A., Langford A.E., Lennox B.R. (2017). Synaptic and neuronal autoantibody-associated psychiatric syndromes: Controversies and hypotheses. Front Psychiatry.

[52] Hansen N., Lipp M., Vogelgsang J., Vukovich R., Zindler T., Luedecke D. (2020). Autoantibody-associated psychiatric symptoms and syndromes in adults: A narrative review and proposed diagnostic approach. Brain Behav Immun Health.

[53] Felblinger J., Slotboom J., Kreis R., Jung B., Boesch C. (1999). Restoration of electrophysiological signals distorted by inductive effects of magnetic field gradients during MR sequences. Magn Reson Med.

[54] Lee T.W., Girolami M., Sejnowski T.J. (1999). Independent component analysis using an extended Infomax algorithm for mixed subgaussian and supergaussian sources. Neural Comput.

[55] Zahneisen B., Hugger T., Lee K.J., LeVan P., Reisert M., Lee H.L. (2012). Single shot concentric shells trajectories for ultra fast fMRI. Magn Reson Med.

[56] Assländer J., Zahneisen B., Hugger T., Reisert M., Lee H.L., LeVan P., Hennig J. (2013). Single shot whole brain imaging using spherical stack of spirals trajectories. Neuroimage.

[57] Cox R.W. (1996). AFNI: Software for analysis and visualization of functional magnetic resonance neuroimages. Comput Biomed Res.

[58] Birn R.M., Diamond J.B., Smith M.A., Bandettini P.A. (2006). Separating respiratory-variation-related fluctuations from neuronal-activity-related fluctuations in fMRI. Neuroimage.

[59] Pijnenburg R., Scholtens L.H., Ardesch D.J., de Lange S.C., Wei Y., van den Heuvel M.P. (2021). Myelo- and cytoarchitectonic microstructural and functional human cortical atlases reconstructed in common MRI space. Neuroimage.

[60] Simmons A., Strigo I., Matthews S.C., Paulus M.P., Stein M.B. (2006). Anticipation of aversive visual stimuli is associated with increased insula activation in anxiety-prone subjects. Biol Psychiatry.

[61] Uddin L.Q. (2015). Salience processing and insular cortical function and dysfunction. Nat Rev Neurosci.

[62] Herken J., Prüss H. (2017). Red flags: Clinical signs for identifying autoimmune encephalitis in psychiatric patients. Front Psychiatry.

[63] De la Peña-Arteaga V., Berruga-Sánchez M., Steward T., Martínez-Zalacaín I., Goldberg X., Wainsztein A. (2021). An fMRI study of cognitive reappraisal in major depressive disorder and borderline personality disorder. Eur Psychiatry.

[64] Peer M., Prüss H., Ben-Dayan I., Paul F., Arzy S., Finke C. (2017). Functional connectivity of large-scale brain networks in patients with anti-NMDA receptor encephalitis: An observational study. Lancet Psychiatry.

[65] Heine J., Prüss H., Kopp U.A., Wegner F., Then Bergh F., Münte T. (2018). Beyond the limbic system: Disruption and functional compensation of large-scale brain networks in patients with anti-LGI1 encephalitis. J Neurol Neurosurg Psychiatry.

[66] Hartung T.J., Bartels F., Kuchling J., Krohn S., Leidel J., Mantwill M. (2024). MRI findings in autoimmune encephalitis. Rev Neurol (Paris).

