# Supplementary material for: Functional Brain Activity Associated With Intermittent Rhythmic Delta/Theta Activity: A Transdiagnostic Electroencephalography–Functional Magnetic Resonance Imaging Resting-State Study
Source: Biol Psychiatry Glob Open Sci. 2025 Nov 29;6(2):100661. doi: 10.1016/j.bpsgos.2025.100661 (PMC12861157; doi:10.1016/j.bpsgos.2025.100661)
Supplement: Table S1 [file mmc1.pdf]

SUPPLEMENTARY INFORMATION

Functional Brain Activity Associated With Intermittent Rhythmic Delta/Theta Activity: A Transdiagnostic Electroencephalography-Functional Magnetic Resonance Imaging Resting-State Study

Feige *et al.*

	**Patients (N=33)**		**Patients (N=33)**
**Antibody findings**			
**Well-characterized anti-CNS antibodies**			
**In CSF**		**In Serum**	
Anti-LGI1<br>Anti-NMDA-R<br>Anti-MOG<br>Anti-Yo<br>Anti-CASPR2<br>Anti-VGCC<br>Anti-TPO<br>ANAs	0 (0 %)<br>1 (3 %)<br>0 (0 %)<br>0 (0 %)<br>0 (0 %)<br>0 (0 %)<br>0 (0 %)<br>1 (3 %)	Anti-LGI1<br>Anti-NMDA-R<br>Anti-MOG<br>Anti-Yo<br>Anti-CASPR2<br>Anti-VGCC<br>Anti-TPO<br>ANAs	0 (0 %)<br>0 (0 %)<br>2 (6 %)<br>1 (3 %)<br>1 (3 %)<br>1 (3 %)<br>2 (6 %)<br>1 (3 %)
**Novel anti-CNS autoantibodies**			
**In CSF**		**In Serum**	
Anti-astrocytic pattern<br>Anti-vessel pattern<br>Anti-granule cell pattern<br>Anti-cytoplasmic pattern<br>Anti-glia cell pattern<br>Anti-axon initial segment pattern<br>Anti-perinuclear pattern<br>Anti-myelin pattern<br>Anti-Purkinje cell pattern<br>Anti-hippocampal pattern	1 (3 %)<br>5 (15 %)<br>4 (12 %)<br>1 (3 %)<br>1 (3 %)<br>1 (3 %)<br>3 (9 %)<br>3 (9 %)<br>2 (6 %)<br>2 (6 %)	Anti-astrocytic pattern<br>Anti-vessel pattern<br>Anti-granule cell pattern<br>Anti-cytoplasmic pattern<br>Anti-glia cell pattern<br>Anti-axon initial segment pattern<br>Anti-perinuclear pattern<br>Anti-myelin pattern<br>Anti-Purkinje cell pattern<br>Anti-hippocampal pattern	1 (3 %)<br>2 (6 %)<br>2 (6 %)<br>2 (6 %)<br>0 (0 %)<br>1 (3 %)<br>2 (6 %)<br>3 (9 %)<br>0 (0 %)<br>0 (0 %)
**Clinical course**			
**Syndrome overall**<br>Predominant affective spectrum syndrome<br>Predominant schizophreniform psychotic syndrome	17* (51 %)<br><br>16** (48 %)	**Clinical state**<br>Acute state<br>(Partial) remission<br>Chronic disease (>2 years)	9 (27 %)<br>12 (36 %)<br>12 (36 %)
*5 patients with (additional) obsessive-compulsive syndromes, 3 patients with additional neurocognitive symptoms.<br>** 8 patients with additional affective syndromes, 1 patient with additional obsessive-compulsive syndrome.			
**Clinical findings**			

	Patients (N=33)		Patients (N=33)
**CSF findings (N=33)**		**Clinical EEG pathologies (N=33)**	
Increased WBC counts	4 (12 %)	Focal slowing	1 (3 %)
Increased albumin quotients (N=32)	10 (30 %)	Intermittent generalized slowing	10 (30 %)
Increased protein concentration	12 (36 %)	Continuous generalized slowing	1 (3 %)
Increased IgG index	2 (6 %)	Epileptic activity	0 (0 %)
OCB in serum	0 (0 %)	**Overall**	12 (36 %)
OCB in CSF	4 (12 %)		
**Overall**	**18 (55 %)**		
**Clinical routine MRI (N=33)**		**Cerebral FDG-PET pathologies (N=22)**	
Non-specific white matter changes	25 (76 %)		
(Chronic) inflammatory lesions	3 (9 %)	Hypermetabolism	5 (23 %)
Global atrophy	5 (15 %)	Hypometabolism	7 (32 %)
Focal atrophy	5 (15 %)	**Overall**	10 (45 %)
Pineal cyst	6 (18 %)		
Others	5 (15 %)		
**Overall**	27 (82 %)		

Supplemental Table 1: Detailed diagnostic findings during the clinical work-up in atoimmune psychiatric syndromes (such a work-up was not performed in borderline personality disorder patients and healthy controls). Only the predominant antibody was mentioned if several antibodies were positive. Abbreviations: ANAs, anti-nuclear antibodies; CASPR2, Contactin-associated protein-like 2; CSF, cerebrospinal fluid; CNS, central nervous system; EEG, electroencephalography; FDG-PET, [^{18}F] Fluorodeoxyglucose positron emission tomography; HC, healthy controls; LGI1, Leucine-rich glioma-inactivated 1; MOG, myelin oligodendrocyte glycoprotein; MRI, magnetic resonance imaging; N, number; NMDA-R, N-methyl-D-aspartate receptor; OCBs, oligoclonal bands; TPO, thyroperoxidase; VGCC, voltage gated calcium channel; WBC, white blood cell.
